# Toward a molecular pathogenic pathway for *Yersinia pestis* YopM

**DOI:** 10.3389/fcimb.2012.00155

**Published:** 2012-12-11

**Authors:** Annette M. Uittenbogaard, R. Lakshman Chelvarajan, Tanya Myers-Morales, Amanda A. Gorman, W. June Brickey, Zhan Ye, Alan M. Kaplan, Donald A. Cohen, Jenny P.-Y. Ting, Susan C. Straley

**Affiliations:** ^1^Department of Microbiology, Immunology, and Molecular Genetics, University of KentuckyLexington, KY, USA; ^2^Lineberger Comprehensive Cancer Center, University of North CarolinaChapel Hill, NC, USA

**Keywords:** YopM, plague, *Yersinia*, chemokine, microarray

## Abstract

YopM is one of the six “effector Yops” of the human-pathogenic *Yersinia*, but its mechanism has not been defined. After delivery to J774A.1 monocyte-like cells, YopM can rapidly bind and activate the serine/threonine kinases RSK1 and PRK2. However, in infected mice, effects of *Y. pestis* YopM have been seen only after 24–48 h post-infection (p.i.). To identify potential direct effects of YopM *in-vivo* we tested for effects of YopM at 1 h and 16–18 h p.i. in mice infected systemically with 10^6^ bacteria. At 16 h p.i., there was a robust host response to both parent and Δ*yopM-1 Y. pestis* KIM5. Compared to cells from non-infected mice, CD11b^+^ cells from spleens of infected mice produced more than 100-fold greater IFNγ. In the corresponding sera there were more than 100-fold greater amounts of IFNγ, G-CSF, and CXCL9, as well as more than 10-fold greater amounts of IL-6, CXCL10, and CXCL1. The only YopM-related differences were slightly lower CXCL10 and IL-6 in sera from mice infected 16 h with parent compared to Δ*yopM-1 Y. pestis*. Microarray analysis of the CD11b^+^ cells did not identify consistent transcriptional differences of ≥4-fold at 18 h p.i. However, at 1 h p.i. mRNA for early growth response transcription factor 1 (*Egr1*) was decreased when YopM was present. Bone marrow-derived macrophages infected for 1 h also expressed lower *Egr1* message when YopM was present. Infected J774A.1 cells showed greater expression of *Egr1* at 1 h p.i. when YopM was present, but this pattern reversed at 3 h. At 6 h p.i., *Cxcl10* mRNA was lower in parent-strain infected cells. We conclude that decreased *Egr1* expression is a very early transcriptional effect of YopM and speculate that a pathway may exist from RSK1 through *Egr1*. These studies revealed novel early transcriptional effects of YopM but point to a time after 18 h of infection when critical transitional events lead to later major effects on cytokine gene transcription.

## Introduction

The human-pathogenic *Yersinia* possess highly similar virulence plasmids that encode a type 3 secretion system (T3SS) for delivery of proteins into mammalian cells (Perry and Fetherston, 1997). Six of these, termed effector *Yersinia* outer proteins (Yops), function to undermine innate defenses by inhibiting cell signaling necessary for phagocytosis, activation of macrophages (MΦs) and effective anti-bacterial responses by polymorphonuclear leukocytes (PMNs) (Viboud and Bliska, 2011). Enzymatic functions and molecular targets are known for five of the effector Yops; but the mechanism for YopM has not been readily revealed. YopM consists almost entirely of leucine-rich repeats (LRRs) that assemble into a curved parallel beta-sheet structure (Evdokimov et al., 2001). The YopM proteins of the three human-pathogenic *Yersinia* species differ in the number of internal repeats and in the sequences of the internal LRRs but have high homology in the N- and C-terminal LRRs as well as the leader sequence recognized by the T3SS and a short C-terminal tail sequence. The pathogenic significance of the variations is not yet known. YopM of *Y. pestis* and some *Y. pseudotuberculosis* is a 46 kDa highly acidic protein with 15 LRRs (Evdokimov et al., 2001). After infection of HeLa epithelioid cells or J774A.1 monocyte (MO)-MΦ-like cells by *Y. pestis*, YopM was shown to traffic to the perinuclear region of the cell via a vesicular pathway, and variable amounts subsequently were found in the nucleus by 3–4 h post-infection (p.i.) (Skrzypek et al., 1998). YopM possesses at least one non-canonical nuclear localization sequence, but no importin could be identified for its uptake mechanism (Skrzypek et al., 2003; Benabdillah et al., 2004); it could enter the nucleus in association with a host molecule to which it binds in the cytosol (Skrzypek et al., 2003).

Pure YopM has been found to spontaneously penetrate eukaryotic cells due to sequences within the N-terminal leader region (Rüter et al., 2010). Although YopM is delivered to mammalian cells by the T3SS, an intriguing hypothesis has been put forth that type 3 secretion involves an extracellular stage for the Yops in which they are located at the bacterial surface between the bacterium and the host cell (Akopyan et al., 2011). Accordingly YopM might enter the cell from this surface-located compartment by using its spontaneous cell-penetration mechanism that triggers uptake at lipid rafts via caveolae (Rüter et al., 2010). Trafficking of pure YopM in HeLa cells follows a timecourse similar to that after infection: it was found in early endosomes after 15–30 min, late endosomes by 30–60 min, and a perinuclear localization by 60 min (Rüter et al., 2010). At 3 h, small amounts also were found in the nucleus (Rüter et al., 2010). The molecules that YopM binds during its trafficking have not been identified, and the extent to which YopM is exposed to the cytosol or partitions into the cytosol is not known. However, it does bind to all known isoforms of two cytosolic serine/threonine kinases, ribosomal protein S6 kinase (RSK or p90RSK) and protein kinase C-like (PRK or PKN) (McDonald et al., 2003; Hentschke et al., 2010); and YopM-associated complexes were found to contain combinations of the various RSK and PRK family members (Hentschke et al., 2010). In association with YopM, the two types of kinases were sequentially activated, with initial activation of RSK1 which in turn activated PRK2 (McDonald et al., 2003). RSK family members lie down-stream of ERK1/2 in the Ras-MAPK pathway. However, YopM did not affect activation of ERK but instead prevented dephophosphorylation of all RSK isoforms, perhaps by shielding them from phosphatases (Hentschke et al., 2010). YopM-RSK1 complexes were obtained within 30 min of infection of RAW 267.4 MO-MΦ-like cells by *Y. pseudotuberculosis* (McCoy et al., 2010), suggesting that such complexes form rapidly in the cytosol after contact between *Yersinia* and a host cell. Although RSK1 does shuttle between cytosol and nucleus, it was found that binding to RSK1 was not required for YopM to localize to the nucleus (McCoy et al., 2010). RSK and PRK do not normally function together; accordingly YopM was proposed to provide a scaffold for assembling a novel complex with novel substrate specificity, because several tested normal targets of these kinases (Bad, Jun, CREB, Akt) were found not to be activated by the presence of YopM (McDonald et al., 2003).

At present, it is not understood how these *in-vitro* observations underlie the effect YopM has in disease. The effect of the absence of YopM on lethality varies considerably with the route of infection and strain of mice that are used. YopM is required for full virulence of wildtype *Y. pestis* CO92 in bubonic plague in C57BL/6 mice, but it is not required for lethality in pneumonic plague (Ye et al., 2011). It is a major virulence determinant for systemic plague caused by conditionally virulent *Y. pestis* KIM5, a popular model strain for studies of plague in BSL2 containment. The KIM5 strain lacks a chromosomally encoded iron-acquisition system needed for virulence from an intradermal route of infection but is fully virulent after intravenous infection of mice (Une and Brubaker, 1984; Perry and Fetherston, 1997). In this model, the absence of YopM results in a modest loss of virulence in outbred Swiss Webster mice (Nemeth and Straley, 1997), whereas virulence is decreased by 4 orders of magnitude in C57BL/6 mice (Kerschen et al., 2004). Accordingly, these mice provide a powerful experimental system in which to dissect the molecular basis of YopM's pathogenic effect. In C57BL/6 mice infected intravenously with doses that were above the LD_50_ of the parent *Y. pestis* KIM5 but below that of Δ*yopM-1 Y. pestis* KIM5-3002, growth of the mutant was curbed in liver and spleen starting at day 2 p.i. (Kerschen et al., 2004; Ye et al., 2009, 2011). This was accompanied by a robust acute inflammatory response involving PMNs and inflammatory dendritic cells (iDCs) that eventually cleared the bacteria (Kerschen et al., 2004; Ye et al., 2009, 2011). In mice infected with the YopM^+^ strain, numbers of iDCs and natural killer (NK) cells began to decline from spleens starting at day 2 p.i., the bacteria continued growing, and the mice ultimately died (Kerschen et al., 2004; Ye et al., 2009, 2011). Concomitantly, levels of mRNA for pro-inflammatory cytokines remained low in spleens of mice infected with YopM^+^ *Y. pestis* but increased strongly in spleens of mice infected with the Δ*yopM-1* strain (Kerschen et al., 2004). Depletion studies implicated iDCs as major cells responsible for controlling growth of Δ*yopM-1 Y. pestis* in spleen and showed that YopM was associated with inhibition of their recruitment (Ye et al., 2009, 2011). These findings prompted the hypothesis that in spleen YopM delivery to MΦs causes downregulated production of chemokines for inflammatory MOs, the cell type that gives rise to iDCs in organs (Ye et al., 2011).

These effects of YopM likely began earlier than day 2 p.i.: data pooled from multiple experiments indicated that the growth deficit of Δ*yopM-1 Y. pestis* was beginning at day 1 p.i. (Ye et al., 2011). We wanted to determine effects of YopM at earlier times to bridge the gap between the first hours of infection studied using *in-vitro* cell models and the previous *in-vivo* studies. The present study was designed to probe for YopM effects on chemokine/cytokine gene expression and protein production in this period, 1 h to 16–18 h p.i.

## Materials and methods

### Bacterial strains and culture

The bacterial strains and plasmids used in this study are listed in Table 1. *Escherichia coli* was used for propagating plasmids that were transferred to *Y. pestis* KIM8-3003.12 as previously described (Forman et al., 2008). All *Y. pestis* strains lacked the ca. 102 bp *pgm* locus specifying the Ybt siderophore-based iron acquisition system that is needed for virulence of *Y. pestis* in peripheral tissues such as skin (Perry and Fetherston, 1997). A strain lacking the *pgm* locus (Δ*pgm*) is conditionally virulent: avirulent in peripheral tissues but essentially fully virulent from an intravenous route of infection. *Y. pestis* was grown at 28°C in supplemented Heart Infusion Broth as described previously (Forman et al., 2008) and was transferred to 37°C for 1 or 3 h, as indicated. Where appropriate, carbenicillin (Cb) was added to cultures to ensure plasmid maintenance.

**Table 1:** **Bacterial strains and plasmids used in this study**.

**Strain**	**Relevant properties**	**Source or references**
***Y. pestis***		
KIM5	Pgm^−^ Lcr^+^; pCD1 pMT1 pPCP1^a^; conditionally virulent Δ*pgm* 2.MED strain; also called KIM D27; YopM^+^ parent strain	Brubaker, 1991
KIM5-3002	Δ*yopM-1* derivative of *Y. pestis* KIM5	Kerschen et al., 2004
KIM8-3003.12	pPCP1^−^ Multiple-Yop mutant; Δ*yopM-1*	Philipovskiy et al., 2005
(KIM8-MYM)	Δ*yopH* Δ*yopE* Δ*yopT* Δ*ypkA* Δ*yopJ*	
***E. coli***		
DH5α	F^−^ ΔΦ80d *lacZ*ΔM15 *endA1 recA1 hsdR17*(r^−^_*m*_ m^+^_*k*_) *supE44 thi-1 gyrA96* Δ (*lacZYA-argF*)*U169; cloning host*	Life technologies
**PLASMIDS**		
pBluescript II SK-	Phagemid cloning vector	Stratagene (Agilent)
pBS10 (pYopM)	*yopM* and native promoter in pBluescript II SK-	Reisner and Straley, 1992
pPCP2::Kan	*kan*-marked pPCP2 (from *Y. pestis* CO92)	Forman et al., 2008

a *The native virulence plasmids of Y. pestis are the 9.6 kb pPCP (encodes the protease Pla), the 70.3 kb Lcr plasmid pCD (encodes the Ysc T3SS and Yops), and the 96.2 kb pFra (also called pMT; encodes the capsular fibril F1) (Perry and Fetherston, 1997)*.

### Infection of mice

All experiments with mice were reviewed and approved by the University of Kentucky Institutional Animal Care and Use Committee. Groups of four 6–8 week old female C57BL/6N.HSD mice (Harlan Sprague Dawley, Inc) were anaesthetized with isoflurane using a rodent anaesthesia machine and infected intravenously in the retro-orbital plexus (RO) with indicated doses as described (Ye et al., 2011). The bacterial doses were confirmed and bacterial burdens in liver and spleen determined by plating serially diluted samples, and splenic leukocytes were isolated, all as done previously (Ye et al., 2011). Two tissue-dissociation methods were used for experiments to obtain CD11b^+^ cells, a Stomacher 80 Lab blender as in previous work (e.g., Ye et al., 2011), and a GentleMACS dissociator (Miltenyi Biotec, Inc.) by using the manufacturer's protocol. The cell populations recovered by these methods were not identical; accordingly, the method used will be specified. In some experiments, livers and spleens from infected mice were fixed in buffered formalin, and paraffin-embedded sections were stained with hematoxylin and eosin. Sections were examined with a Nikon Eclipse E800 microscope and representative areas photographed with a Photometrics CoolSNAP cf camera.

### Isolation of CD11b^+^ cells

Splenic leukocytes were depleted of T cells, B cells, and PMNs by immunomagnetic beads (Miltenyi), with amounts and procedures as recommended by the manufacturer's protocol. This process also removed dead cells, which bind non-specifically to the beads. CD11b^+^ cells were then recovered on anti-CD11b antibody-coupled immunomagnetic beads. The manufacturer has verified that these beads will recover macrophages from spleen. Briefly, leukocytes from four spleens per infecting bacterial strain were pooled in ice-cold cell-column buffer (PBS pH 7.2, 0.5% BSA, 2 mM EDTA). The cells were pelleted and resuspended in the buffer, and biotinylated anti-Ly6G was added. After gentle mixing by inversion on a tube rotator for 10 min at 4–8°C, microbeads conjugated with anti-CD5, anti-CD19, or anti-biotin antibodies were added, the cell suspensions were mixed on the rotator for 15 min at 4–8°C, washed by centrifugation, and resuspended in 1 mL cell-column buffer. The suspensions were applied to LS separation columns (Miltenyi) at room temperature, and flowthrough cells (containing CD11b^+^ cells) were collected through a 20 gauge needle into tubes on ice. The columns were washed with cold buffer, giving a total effluent of 13 mL. The collected cells were centrifuged and resuspended in 0.1 mL buffer per column. Samples of diluted cells were stained with trypan blue for viability assessment and were counted in a hemocytometer. The cells were then positively selected for CD11b^+^ cells with anti-CD11b-conjugated microbeads in combination with a MACS Separator to retain anti-CD11b bead-labeled cells prior to flushing the cells off the column. This process typically yielded 1–2 × 10^7^ cells per pool from four mice, consistent with CD11b^+^ cells constituting ca. 5% of the splenic leukocytes (Ye et al., 2011), and the preparations consistently contained >95% viable cells. After removal of samples for differential cell counting and flow cytometry, the remaining cells were suspended in RLT/BME (Qiagen) following the manufacturer's protocol. They were snap-frozen in liquid N_2_ and stored at −80°C.

### Analysis of cell populations

Differential counts for major leukocyte types were made for cell populations that were recovered following immune-magnetic bead treatment. Slides were prepared with a cytocentrifuge, stained with Giemsa, and cells scored from randomly selected fields. For the RNA microarray experiments, between 140 and 611 cells were scored per sample; for the multiplex chemokine experiments, between 570 and 972 cells were scored. The cells analyzed by RNA microarray also were analyzed for presence of Ly6G, Cd11b, Gr1, and CD11c (the 1-h infections) or Gr1 and F4/80 (the 18-h infections) by flow cytometry. Cell preparation for flow cytometry was done as previously described (Ye et al., 2011), and the analysis was carried out using a BD Biosciences LSRII flow cytometer. Dead cells and debris were gated using ethidium monoazide (EMA, Sigma) viability staining. The data were analyzed by FlowJo software (Version 7.6.1; Tree Star). Antibodies used were from BD Pharmingen, Inc.: fluorescein isothiocyanate (FITC)-conjugated anti-CD11b, allophycocyanin (APC)-conjugated F4/80, phycoerythrin (PE)-conjugated anti-Ly6G, peridinin chlorophyll protein-cyanine tandem dye (PerCP-Cy5.5)-conjugated anti-Gr1, PE-cyanine tandem dye (PE-Cy7)-conjugated anti-CD11c, and Fc Block™ (Rat Anti-Mouse CD16/CD32).

### Multiplex MAP cytokine/chemokine analysis

This set of experiments used the Miltenyi GentleMACS dissociator to dissociate spleens. Chemokines and cytokines made in response to 1 or 16 h infection with parent and Δ*yopM-1 Y. pestis* were analyzed by Milliplex MAP chemokine array that assays 22 inflammation-related cytokines and chemokines. The experimental series included several control and reference treatments as well as serum samples from some mice. (1) The CD11b^+^ cells from mice infected for 1 or 16 h and from non-infected control mice were made to 2 × 10^6^ cells ml^−1^ in RPMI + 10% FBS and cultured for 6 h in 12-well dishes at 37°C, 5%CO_2_. Culture supernatants containing secreted proteins were filtered through a 0.45 μm pore-size low-protein-binding filter and snap-frozen. (2) Serum was collected from all 16 h-infected mice and from non-infected mice, pooled in equal ratio from the four mice per group, filtered, and snap-frozen. (3) Non-fractionated splenic leukocytes from mice infected 1 h and from non-infected mice were cultured, filtered, and frozen as above. For the non-infected splenocytes, duplicate sets of wells contained 20 ng ml^−1^ phorbol myristyl acetate (PMA). (4) Non-fractionated splenic leukocytes from mice given 25 μg *E. coli* O111:B4 LPS (Sigma) by the RO route were obtained 1 h later and cultured, filtered, and frozen as for the CD11b^+^ cells. All of these experiments were carried out in triplicate on different days, using 108 mice total. The chemokine array analysis was performed by Millipore/Merck Biomarker Services (EMD Millipore, a division of Merck KGaA).

### RNA microarray analysis

This set of experiments used the Stomacher 80 Lab blender to dissociate spleens. Total RNA was obtained from CD11b^+^ cells stored in RNAlater (Qiagen) by using the RNeasy mini-prep kit per the manufacturer's instructions (Qiagen). RNA integrity was assessed by comparing 18S and 28S rRNA species using gel electrophoresis on a Bioanalyzer (Agilent Technologies, Inc.). RNAs from duplicate experiments (each using four mice per infecting *Y. pestis* strain) were pooled 1:1 (except for 1-h infection with ΔyopM-1 Y. pestis, where triplicate experiments were used and the RNAs were pooled in equal ratios), and the pools were divided into three aliquots which were handled independently to generate three hybridization data sets. Fluorescent-labeled Cy5 (red) aRNA sample probes were prepared by reverse transcription with SuperScriptIII reverse transcriptase (Invitrogen), 1-round of amplification with amino-allyl nucleotides according to manufacturer's protocol (Epicentre Biotechnologies) and coupling to Cy5 (red) dye (GE Healthcare). Likewise Cy3 (green) labeled aRNA probe was prepared using the Universal mouse reference RNA comprised of RNA pooled from 11 mouse cell lines (Stratagene). Each Cy5 sample aRNA probe was paired with Cy3 reference aRNA probe and hybridized to microarrays in formamide-containing buffer overnight at 42°C using a Maui Hybridization System (BioMicro Systems Inc.). The custom-designed microarray consisted of 70-mer oligonucleotides representing 1555 immune response gene targets. Commercially available and custom-designed oligos were purchased from Operon Biotechnologies, Inc. and printed onto glass slides by the Duke Microarray Facility (Duke University, Durham, NC). Each microarray contained replicate targets spotted four times. The gene target pool also included housekeeping genes (i.e., Actb, Gapd, Tuba, ribosomal proteins Rpl13a, Rpl18, Rps9) used as internal references, negative background controls and known “spiked-in” RNA oligos (Applied Biosystems). The hybridized spot intensities were acquired using a GenePix 4000B scanner (Molecular Devices Corp) and assessed using GenePix Pro 6.0 software. Median pixel intensities (with background subtracted) were uploaded in the GeneSpring X software package (Agilent Technologies, Inc), normalized by global LOWESS fit (Cleveland, 1979), log base 2 transformed, filtered such that probes exceeding the background and spot quality criteria (i.e., “absent” probes) were not included and merged into datasets. Gene lists and hierarchical clusters of differentially expressed genes were generated. R software through Bioconductor (http://CRAN.R-project.org) was employed as another approach to analyze hybridization signal intensities (Becker et al., 1988; Zhang et al., 2009). The normalized log ratio expression data from the GeneSpring X analysis for all the genes in any sample that met the filtering criteria of “flags” on the microarray called present or marginal, but not absent, and for which the signal was above background in at least 3 of the 12 samples have been deposited in NCBI's Gene Expression Omnibus (Edgar et al., 2002) and are accessible through GEO Series accession number GSE41564 (http://www.ncbi.nlm.nih.gov/geo/query/acc.cgi?acc=GSE41564).

### Quantitative RT-PCR confirmation and evaluation

Several gene targets were analyzed on the RNA used for microarray as well as from additional replicate experiments by two-step quantitative RT-PCR (qRT-PCR) using SYBR Green detection and quantification in a LightCycler 2.0 instrument equipped with version 4.0 software (Roche). Primer sequences were obtained from the RTPrimer Database (www.rtprimerdb.org).

### Tissue-culture infection models

Bone marrow-derived macrophages (BMMs) were used after 7–10 days culture of femoral bone marrow in 12-well dishes in medium comprised of 60% Dulbecco's Modified Eagle Medium (high glucose supplemented with glutamine and HEPES; GIBCO 11960: Life Technologies, Inc.) 20% heat-inactivated FBS (GIBCO 26140-079: Life Technologies), 20% filtered L929 cell-conditioned medium, and 1% penicillin-streptomycin-neomycin mixture (GIBCO 15640: Life Technologies). J774A.1 mouse macrophage-like cells (ATCC) were cultured in 24-well dishes in RPMI 1640 (supplemented with glutamine and HEPES: Mediatech, Inc. 10-041-CV: Corning Life Sciences) + 10% heat-inactivated FBS. Both cell types were grown to ~95% confluence and rested for 3 h in medium without FBS prior to infection. *Y. pestis* that had been grown to an OD_620_ of ca. 1.0 were pelleted and resuspended to give the desired MOI (10–40) in the appropriate tissue-culture medium without FBS and incubated, 1 ml per well, in 12-well plates at 37°C + 5% CO_2_. Medium for uninfected control wells was similarly incubated. After 1–3 h, the medium over the BMMs or J774A.1 cells was replaced by bacteria-containing or control medium, and the infection was initiated by a 5-min centrifugation. After infection for 1–6 h, the medium was replaced by 1 mL RNAlater (Qiagen) per well, and RNA was isolated by following the protocol for Qiagen RNeasy Mini Kit for adherent cells.

### Statistics

For genomic analyses, a right-tailed Fisher's Exact Test was employed to produce a significance *p*-value that described how likely genes from a specific dataset participated in a desired function. For other assays, significance of differences was evaluated with the two-tailed unpaired Student's *t* test.

## Results

### High-dose systemic plague model

This project was undertaken to characterize early mRNA and cytokine responses that lead to the transcriptional and cellular recruitment differences seen at 2 days p.i. (Kerschen et al., 2004; Ye et al., 2011). We developed a high-dose intravenous infection model to provide a detectable host response at these early times. This route gives a synchronous infection in which the bacteria seed spleen and liver within 30 min (Conlan, 1997; Burnett et al., 2004). The dose of 10^6^ potentially would provide immediate interactions with a significant fraction of immune cells known to be targeted by *Y. pestis*: MΦs, MOs, PMNs, and DCs (Lukaszewski et al., 2005; Marketon et al., 2005). Prior to infection, the bacteria were given a 3-h incubation at 37°C to induce thermally-upregulated genes such as those for the T3SS and Yops, because during the natural infection yersiniae that reach internal organs would have been pre-exposed to mammalian body temperature.

**Figure 1:**
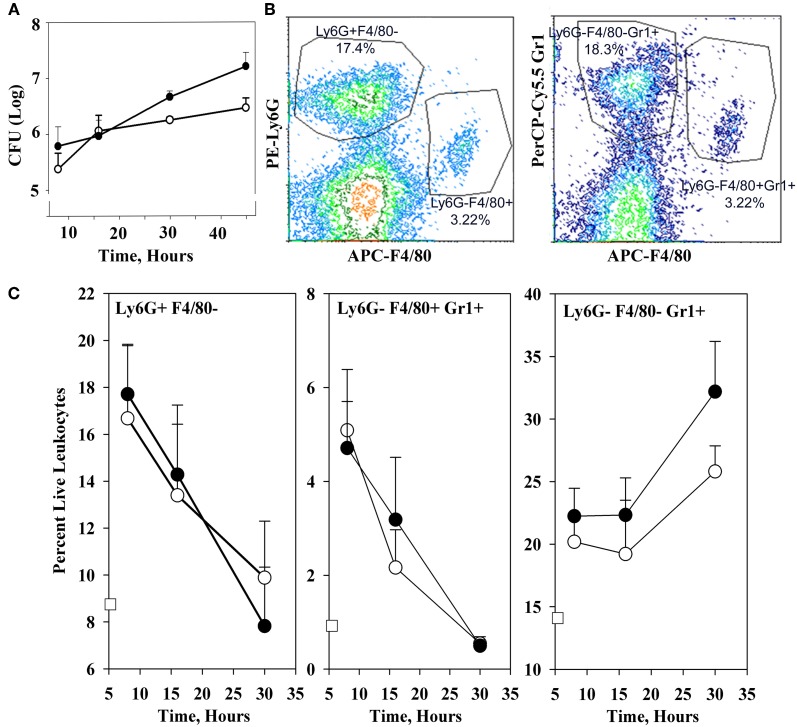
**Infection dynamics and host cellular responses in spleen in the high-dose systemic plague model**. C57BL/6 mice were infected intravenously with a dose of 10^6^ of the parent *Y. pestis* KIM5 (closed circles) or the Δ*yopM-1Y. pestis* KIM5-3002 (open circles) that had been induced for expression of thermally-upregulated properties for 3 h at 37°C. Bacterial burdens were determined as CFU Panel **(A)**. Bacterial numbers differed significantly for parent and Δ*yopM-1 Y. pestis* at 30 and 45 h p.i. (*P* <10^−4^ and *P* < 10^−2^, respectively). Splenic leukocytes were stained with fluorochrome-conjugated antibodies against the surface markers Ly6G, F4/80, and Gr1 as well as the dye EMA which stains cells that have lost membrane integrity, and analyzed by flow cytometry. Panel **(B)** illustrates the gating strategy used to define and quantify several host cell populations as percent live leukocytes. Panel **(C)** shows how the percent of EMA^−^ (i.e., “live”) leukocytes changed over 30 h of infection for three inflammatory cell types of relevance to the host response to *Y. pestis*. Values for non-infected mice are given by the open squares on the ordinates. Percentages of Ly6G^+^ F4/80^−^ PMNs at 16 h were significantly different from those at 8 h for mice infected with the Δ*yopM-1* strain (*P* < 0.05 by student's *t*-test); those at 30 h differed significantly from 8 h for infections by both *Y. pestis* strains (*P* < 10^−2^ for parent and *P* < 10^−4^ for Δ*yopM-1*). For Ly6G^−^ F4/80^+^ Gr1^+^ MOs, percentages at both 16 and 30 h differed significantly from those at 8 h for both infections (parent: *P* < 0.05 for 16 h, *P* < 10^−5^ for 30 h; Δ*yopM-1*: *P* < 10^−2^ for 16 h, *P* < 10^−5^ for 30 h). For the Ly6G^−^ F4/80^−^ Gr1^+^ population, percentages differed only for 30 compared to 8 h p.i. (*P* < 10^−2^ for parent *Y. pestis* and *P* < 10^−3^ for Δ*yopM-1*). Each datum point gives the averages ± standard deviation (SD) for pooled data from six mice in two independent experiments.

**Figure 2:**
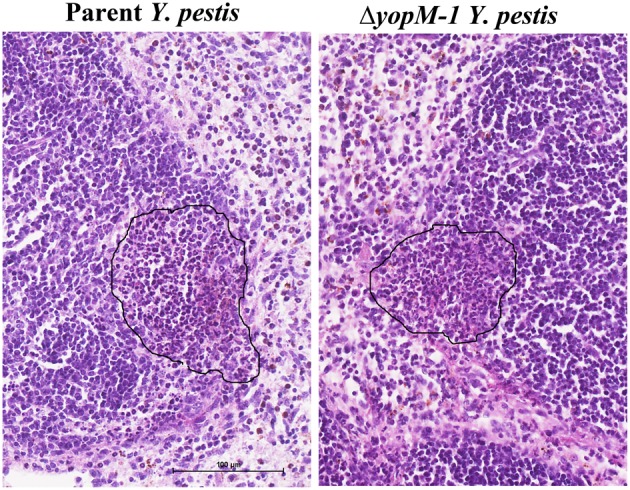
**Histopathology of spleen at 16 h p.i. C57BL/6 mice were infected for 16 h as in the experiments of Figure 1, and spleens were fixed in formalin and stained with hematoxylin and eosin**. Representative sections are shown. Left panel, spleen from a mouse infected with parent *Y. pestis*; Right panels, spleen from a mouse infected with Δ*yopM-1 Y. pestis*. In each panel the surround indicates an inflammatory focus at the edge of a lymphoid follicle.

Figure 1A shows that the YopM-associated growth difference was manifested as a 10-fold difference in bacterial burden at 30 h p.i., demonstrating that a YopM-related host-response phenotype was present in this infection model. Up to 16 h p.i., the two *Y. pestis* strains grew at the same rate. This absence of a difference in bacterial burden at 16 h has important implications which will be discussed later. By 16 h, the host response to infection had clearly initiated, based on the presence of inflammatory foci in spleens of mice infected with either *Y. pestis* strain (Figure 2). These were located at the periphery of the follicles and adjacent red pulp. The live inflammatory cells in infected spleens were characterized by flow cytometry. Figure 1B illustrates the gating strategy used to define and quantify these cells, and Figure 1C shows the dynamics of three populations during the development of disease: Ly6G^+^ F4/80^−^ mature PMNs, Ly6G^−^ F4/80^+^ Gr1^+^ MOs, and Ly6G^−^ F4/80^−^ Gr1^+^ cells, comprised of iDCs, plasmacytoid DCs, some CD8^+^ T cells, and myeloid-derived suppressor cells (MDSCs) (Kung et al., 1991; Egan et al., 2008; Auffray et al., 2009). By 8 h p.i., all three populations had markedly increased in prevalence over the levels present in non-infected mice (open squares on the ordinates). However, by 16 h p.i., and further at 30 h, the PMNs and MOs had declined in prevalence in both infections. In contrast, the net percentages of the heterogeneous Ly6G^−^ F4/80^−^ Gr1^+^ population remained the same at 16 h as at 8 h p.i.. By 30 h the mice were clearly sick, and the decreases in other populations resulted in an apparent increase in percentage of Ly6G^−^ F4/80^−^ Gr1^+^ cells. We did not investigate the basis of inflammatory cell loss but note that it is well known that initially acute inflammatory foci evolve into cell-poor lesions at later times in systemic plague even for Δ*yopM-1 Y. pestis* when the bacterial numbers are similar to those in our study Ye et al. (2011). Based on these observations, we elected to restrict our studies to around 16 h p.i., and earlier, when there were no differences in bacterial burdens between infecting strains that could influence the host response in a non-YopM-related manner. At 16 h, the bacteria were replicating, and a robust host cellular response was underway. This time point would be long enough after infection that the influence of YopM on gene expression in inflammatory cells as well as resident cells could be manifested. As a very early point in infection we chose 1 h p.i., when inflammatory foci were not detected (data for liver not shown) and the bacteria likely were interacting mainly with resident cells.

### Cytokines and chemokines in splenic cells and sera of mice infected with YopM^+^ and YopM^−^ *Y. pestis*

Putative target cells for YopM in spleens of mice infected for 1 or 16 h were characterized for their release of 22 chemokines and cytokines after a 6-h incubation *in-vitro*. We wanted to remove non-critical cells from the analysis in an effort to focus on cells that mediate the pathogenic effect of YopM and thereby increase the signal-to-noise ratio. Because mice lacking T cells and B cells or depleted of Ly6G^+^ PMNs still control growth of Δ*yopM-1 Y. pestis* in spleen as efficiently as wild-type mice (Kerschen et al., 2004; Ye et al., 2011), these cells are not critical cells for the pathogenic effect of YopM (or their absence would allow the Δ*yopM-1* strain to grow like the YopM^+^ parent strain). Accordingly, we removed B cells, T cells, and Ly6G^+^ PMNs from splenic leukocytes of infected mice and selectively recovered CD11b^+^ cells for chemokine assays. These included resident MΦs and myeloid DCs (mDCs), MOs, any iDCs present at 16 h, and MDSCs as well as immature PMNs not removed by the anti-Ly6G-coupled beads (Frazer et al., 2011). In addition, cytokine production by total splenic leukocytes was assayed for mice infected 1 h, and sera were analyzed from mice infected for 16 h. Two classic stimulatory treatments were included as systems controls: mice were treated with LPS for 1 h and their total splenic leukocytes were assayed for production of chemokines, and total splenic leukocytes from non-infected mice were treated with PMA during the 6-h *in-vitro* culture period. These assays were needed to validate the duration of culturing for chemokine/cytokine production and the ability to detect host responses after only 1 h of stimulus.

Table 2 gives the percentage of cell types recovered from the anti-CD11b-conjugated beads after 1 or 16 h infection with parent or Δ*yopM-1 Y. pestis*. Surprisingly, the recovered populations were dominated by comparable numbers of MO/MΦs and immature PMNs (ring forms, band cells, and intermediate stages between these). It cannot be ruled out that some of these were MDSCs, which also are CD11b^+^ Gr1^+^ cells present in spleens of healthy mice (Ribechini et al., 2010). These cells have been documented to function in suppression of T cell responses (Ribechini et al., 2010) and, in one case, resolution of infection (Poe et al., 2012). In the present study the cells were obtained during the early phase of acute inflammation and most likely were immature PMNs.

**Table 2:** **Distribution of cell types among splenic CD11b^+^ cells analyzed by multiplex chemokine/cytokine array**.

**Treatment**	**MO/MΦ**	**PMN**	**Other**	**Lymphocytes**
**Non-infected mice**	43 ± 8	36 ± 4	1 ± 0	19 ± 4
**MICE INFECTED 1 h**				
Parent *Y. pestis*	41 ± 6	36 ± 5	4 ± 3	21 ± 3
Δ*yopM-1 Y. pestis*	46 ± 6	31 ± 2	1 ± 0	22 ± 8
**MICE INFECTED 16 h**				
Parent *Y. pestis*	45 ± 5	42 ± 8	1 ± 0	12 ± 6
Δ*yopM-1 Y. pestis*	45 ± 6	45 ± 7	1 ± 0	10 ± 2

There were no significant differences in prevalence of cell types between mice infected with YopM^+^ and Δ*yopM-1 Y. pestis* for either infection time. Although there was a trend toward increased prevalence of PMNs at 16 h compared to cells from 1 h p.i., and non-infected mice, this effect was not statistically significant. These findings are consistent with our previous observations that MOs accumulate in *Y. pestis*-infected spleens alongside or even before the PMNs and that up to day 1 p.i. the numbers of both cell types (as assayed by flow cytometry) do not differ between mice infected with the two *Y. pestis* strains (Ye et al., 2009).

Table 3 shows that non-infected splenocytes cultured for 6 h with PMA produced detectable amounts of nine chemokines in all three experiments, in contrast to ones cultured with medium alone, which produced small amounts only of CCL5 and, in two experiments, of CXCL9 and CXCL10. Splenocytes from mice treated with LPS for 1 h consistently produced twelve of the chemokines/cytokines during subsequent culture with medium alone, and these generally were present in significantly greater amounts than from splenocytes stimulated *in-vitro* with PMA. These reference tests showed that the test system functioned well and had the potential to provide meaningful results with cells from infected mice.

**Table 3:** **Control tests for multiplex analysis of chemokines and cytokines^a^^,^^b^**.

	**Cytokine/chemokine concentration, pg ml^−1^**						
**Treatment**	**G-CSF**	**GM-CSF**	**TNFα**	**IL-1β**	**IL-6**	**CXCL1**	**IFNγ**
**MEDIUM-ONLY CONTROLS**							
With 20 ng ml^−1^ PMA	<3	<16	<3	<16	<16	<3	<3
Medium only	<3	<16	<3	<16	<16	<3	<3
**SPLENOCYTES**							
**Non-infected mice**							
Incubated with 20 ng ml^−1^ PMA	<3	28 ± 9	13 ± 5	<16	–^c^	<3	5 ± 1
Incubated without PMA	<3	<16	<3	<16	<16	<3	<3
**Mice given LPS 1 h**							
1 mg/kg *E. coli* LPS given RO	6 ± 1	–^c^	27 ± 3	100 ± 37	133 ± 35	19 ± 2	43 ± 10
**Mice infected 1 h**							
Parent *Y. pestis*	–^c^	<16	158 ± 148^d^	–^c^	–^c^	–^c^	<3
Δ*yopM-1 Y. pestis*	5 ± 0^d^	<16	83 ± 35^d^	–^c^	77 ± 13^d^	5 ± 2^d^	<3
	**CCL5**	**CXCL9**	**CXCL10**	**CXCL2**	**VEGF**	**IL-10**	**IL-17**
**MEDIUM-ONLY CONTROLS**								
With 20 ng ml^−1^ PMA	<3	<16	<3	<80	<3	<3	<3
Medium only	<3	<16	<3	<80	<3	<3	<3
**SPLENOCYTES**								
**Non-infected mice**							
Incubated with 20 ng ml^−1^ PMA	96 ± 48	76 ± 23	35 ± 16	243 ± 73	8 ± 4	19 ± 17	–^c^
Incubated with medium only	11 ± 3	33 ± 8^d^	7 ± 4^d^	<80	<3	–^c^	<3
**Mice given LPS 1 h**							
1 mg/kg *E. coli* LPS given RO	154 ± 17	228 ± 55	288 ± 46	156 ± 34	–^c^	27 ± 5	9 ± 2
**Mice infected 1 h**							
Parent *Y. pestis*	44 ± 20	100 ± 35	33 ± 11	–^c^	<3	13 ± 6^d^	–^c^
Δ*yopM-1 Y. pestis*	45 ± 24	103 ± 34	48 ± 26	428 ± 218^d^	<3	8 ± 2^d^	–^c^

a *<signifies that data from all three experiments were below the detection limit. Other values without a superscript are average ± SD based on data that were above the detection limit in all three experiments*.

b *Results for IL-4, IL-12(p40), IL-12(p70), IL-15, Eotaxin, CXCL5, CCL2, and M-CSF are not listed because all samples returned responses below the detection limit for these chemokines/cytokines*.

c *–denotes that there was only one response above the detection limit; the other two experiments returned data that were below the detection limit*.

d *Average ± the range from two experiments; the third experiment returned data that were below the detection limit*.

Table 4 shows the chemokine/cytokine profiles for sera and CD11b^+^ cells from these experiments. The analyses of sera from mice infected with both YopM^+^ and Δ*yopM-1 Y. pestis* confirmed that a robust cytokine/chemokine response was underway by 16 h p.i., with levels of G-CSF and CXCL9 greater than 100-fold above those in non-infected mice, and high levels of IL-6, CXCL1, CCL2, CXCL10, and IFNγ. Curiously, GM-CSF and IL-1β were not detectable in sera. Six chemokines (including TNFα) were present at levels less than 5-fold above those in non-infected mice, and amounts of five others, including IL-4 and IL-10, were comparable to those in non-infected mice. Of the 22 chemokines and cytokines assayed in sera, two differed between mice infected with the two *Y. pestis* strains: CXCL10 (significant at *P* = 0.0423) and IL-6 (not quite significant at *P* = 0.066), but the differences were less than 2-fold. Overall, these experiments revealed that mice infected with a large dose of *Y. pestis* produced only a marginal systemic cytokine response at 1 h p.i., but a strong and selective systemic response that was independent of the presence of YopM in the infecting strain by 16 h p.i.

**Table 4:** **Chemokines and cytokines from sera and splenic CD11b^+^ cells of mice infected with YopM^+^ and Δ*yopM-1 Y*. *pestis*^a^**.

	**Cytokine/chemokine concentration, pg ml^−1^**							
**Treatment**	**G-CSF**	**GM-CSF**	**TNFα**	**IL-1β**	**IL-6**	**CXCL1**	**IFNγ**	
**Sera**								
**Non-infected mice**	452 ± 93	<160	10 ± 1	<160	<32	74 ± 18	20 ± 8	
**Mice infected 16 h**								
Parent *Y. pestis*	97,401 ± 27,167	<160	43 ± 5	<160	1341 ± 485	2087 ± 231	7291 ± 3042	
Δ*yopM-1 Y. pestis*	111,939 ± 42,016	<160	48 ± 5	<160	2476 ± 413	2351 ± 869	6876 ± 1326	
**CD11b^+^ CELLS**								
**Non-infected mice**	<3	<16	38 ± 19	<16	33 ± 3	7 ± 2	<3	
**Mice infected 1 h**								
Parent *Y. pestis*	10 ± 3^c^	<16	149 ± 139^c^	<16	–^b^	19 ± 14^c^	<3	
Δ*yopM-1 Y. pestis*	–^b^	<16	53 ± 65	<16	–^b^	8 ± 4^c^	<3	
**Mice infected 16 h**								
Parent *Y. pestis*	–^b^	22 ± 2^c^	27 ± 27	48 ± 22	150 ± 155	23 ± 12	1712 ± 1040	
Δ*yopM-1 Y. pestis*	–^b^	<16	373 ± 519	48 ± 16^*^	1076 ± 1478	41 ± 25	650 ± 249^*^	
	**CCL5**	**CXCL9**	**CXCL10**	**CXCL2**	**VEGF**	**IL-10**	**IL-17**	
**SERA**								
**Non-infected Mice**	54 ± 4	410 ± 53	220 ± 14	<800	<6	<32	8 ± 0	
**Mice infected 16 h**								
Parent *Y. pestis*	80 ± 35	76,612 ± 3276	3116 ± 186	1151 ± 281^c^	28 ± 10	–^b^	11 ± 6	
Δ*yopM-1 Y. pestis*	88 ± 16	50,585 ± 14,345	3709 ± 215^**^	969 ± 86^c^	23 ± 0^c^	<32	15 ± 2	
**CD11b^+^ CELLS**								
**Non-infected mice**	123 ± 73	335 ± 256	391 ± 225	–^b^	<3	–^b^	<3	
**Mice infected 1 h**								
Parent *Y. pestis*	49 ± 25	168 ± 63	235 ± 156	–^b^	<3	<3	<3	
Δ *yopM-1 Y. pestis*	35 ± 8	117 ± 14	217 ± 85	–^b^	<3	<3	<3	
**Mice infected 16 h**								
Parent *Y. pestis*	31 ± 16	1609 ± 868	1112 ± 660	<80	<3	<3	–^b^	
Δ*yopM-1 Y. pestis*	74 ± 82	4373 ± 2751	2127 ± 1521	–^b^	<3	–^b^	7 ± 4^c^	
	**IL-4**	**IL-12(p40)**	**IL-12(p70)**	**IL-15**	**Eotaxin**	**CXCL5**	**CCL2**	**M-CSF**
**SERA**								
**Non-infected mice**	9 ± 1^c^	<32	139 ± 32	<32	568 ± 47	7994 ± 631	76 ± 5	8 ± 2
Parent *Y. pestis*	–^b^	86 ± 28	267 ± 142	942 ± 1188	1584 ± 47	7660 ± 450	2047 ± 237	44 ± 10^c^
Δ*yopM-1 Y. pestis*	–^b^	81 ± 13^c^	262 ± 19	54 ± 19^c^	1537 ± 154	7756 ± 536	1804 ± 245	37 ± 4
CD11b^+^ cells^d^	<3	<16	<3	<16	<3	<400	<16	<3

a *<signifies that data from all three experiments were below the detection limit. Other values without a superscript are average ± SD based on data that were above the detection limit in all three experiments*.

b *–denotes that there was only one response above the detection limit; the other two experiments returned data that were below the detection limit*.

c *Average ± the range from two experiments; the third experiment returned data that were below the detection limit*.

d *CD11b^+^ cells from all treatment groups returned responses below the detection limit for this set of genes, with the exception of mice infected for 16 h with parent Y. pestis, for which a single experiment returned a value of 670 pg ml^−1^ for CXCL5*.

* *Significantly greater than the response from CD11b^+^ cells from non-infected mice (P < 0.05)*.

** *Data from mice infected with the two Y. pestis strains differed significantly at P < 0.05*.

At 1 h p.i., CD11b^+^ cells produced detectable amounts of 7 chemokines/cytokines, but none of these was consistently found at levels above those for cells from non-infected mice. At 16 h p.i., the CD11b^+^ cells produced these same chemokines/cytokines more consistently; but due to the range of responses in the three experiments, only IL-1β and IFNγ (for Δ*yopM-1 Y. pestis*) levels were significantly higher than for CD11b^+^ cell responses from non-infected mice (at *P* < 0.05). There were no significant YopM-related differences in chemokine/cytokine production by CD11b^+^ cells at 16 h p.i.

### Transcriptional profiling of splenic CD11b^+^ cells from mice infected with YopM^+^ and Δ*YopM Y. pestis*

**Figure 3:**
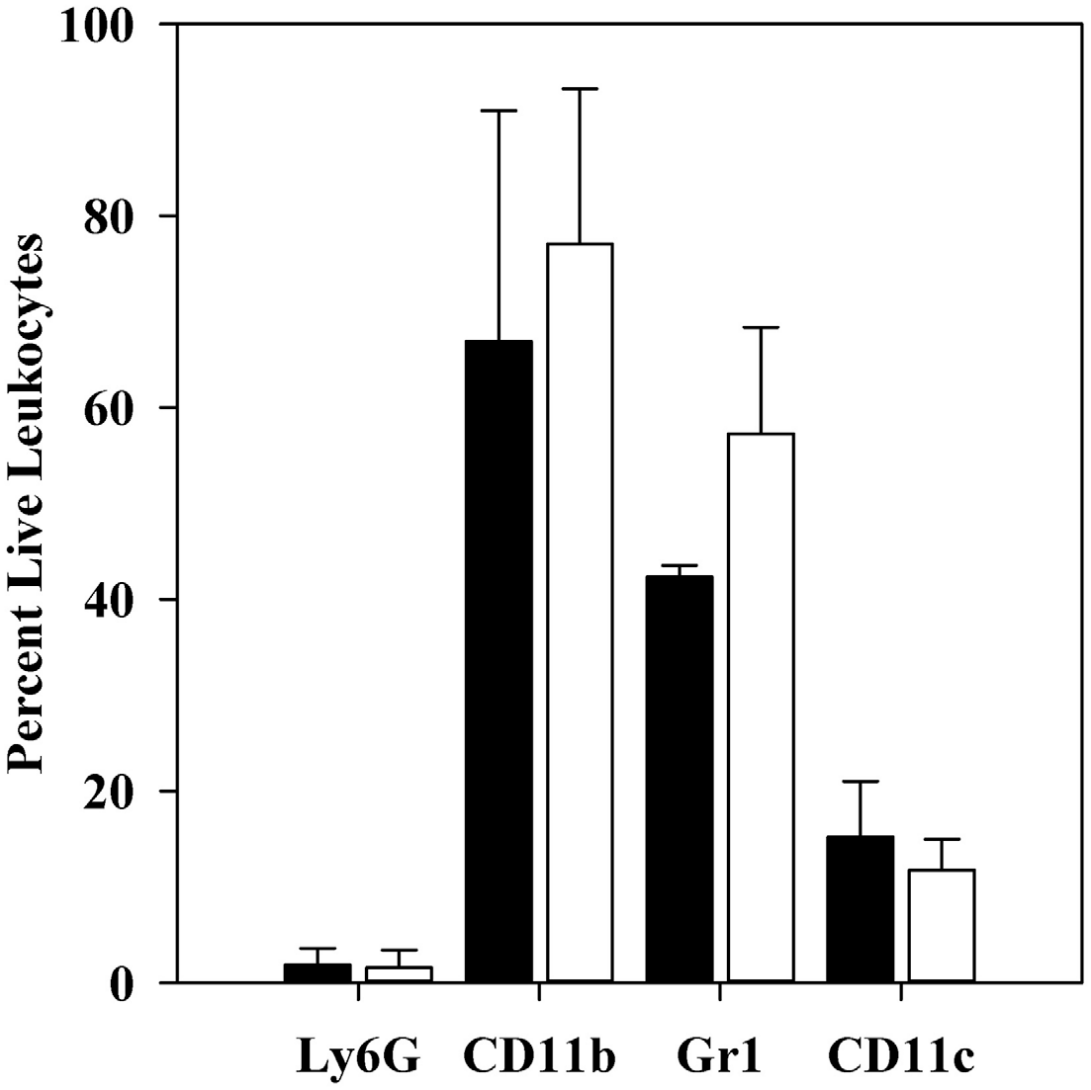
**Flow cytometric analysis of CD11b^+^ cell populations after 1 h infection**. C57BL/6 mice were infected for 1 h with 10^6^ thermally-induced parent or Δ*yopM-1 Y. pestis*, and CD11b^+^ cells were obtained from spleens as described in the legend to Figure 2. Samples were analyzed by flow cytometry for the presence of Ly6G, CD11b, Gr1, CD11c, and for staining by EMA. The bars represent the averages ± SD of the percent live leukocytes. Solid bars, mice infected with parent *Y. pestis* (pooled data from two experiments and 8 mice); open bars, mice infected with Δ*yopM-1 Y. pestis* (pooled data from three experiments and 12 mice).

Levels of mRNAs for a custom panel of immune response genes were measured for CD11b^+^ cells obtained from spleens of infected mice and compared to transcript levels in a commercially produced “Universal mouse reference RNA.” In these experiments, spleens were dissociated with a Stomacher lab blender, producing a cell population skewed toward MOs/MΦs rather than immature PMNs (see Table 5). Figure 3 shows that the majority of these cells were in fact CD11b^+^.

**Table 5:** **Distribution of morphological types among the CD11b^+^ cells used for transcriptional analysis**.

**Treatment**	**MO/MΦ**	**PMN**	**Other**	**Lymphocytes**
**MICE INFECTED 1 h**				
Parent *Y. pestis*	72 ± 3	15 ± 6	0 ± 0	13 ± 3
Δ*yopM-1 Y. pestis*	77 ± 11	10 ± 7	1 ± 1	12 ± 5
**MICE INFECTED 18 h**				
Parent *Y. pestis*	61^a^	20^a^	4^a^	15^a^
Δ*yopM-1 Y. pestis*	66 ± 8	20 ± 12	2 ± 0	14 ± 3

a *Data from one of the experiments done to obtain RNA for microarray analysis; data for ΔyopM-1 Y. pestis were pooled from three experiments. The 1-h data came from three experiments for ΔyopM-1 Y. pestis and 2 for the parent Y. pestis KIM5*.

Also as expected, very few were positive for the Ly6G marker on mature PMNs. The majority (40–60%) of the population was also positive for Gr1 (not significantly different for mice infected by YopM^+^ vs. Δ*yopM-1Y. pestis*). Similar percentages of Gr1^+^ cells were present among the CD11b^+^ cells obtained at 18 h p.i. [53 ± 7 and 63 ± 10% for mice infected with parent and Δ*yopM-1 Y. pestis*, respectively (determined by flow cytometric analysis for Gr1 and F4/80)]. These cells likely were mainly MOs, iDCs, mDCs, and possibly MDSCs, based on the magnetic bead selections that produced them. About 10% of the cells were positive for the DC marker CD11c.

Table 6 gives the microarray findings for genes expressed ≥2-fold higher or lower by splenic CD11b^+^ cells from mice infected 1 h with parent vs. Δ*yopM-1 Y. pestis*. As expected for this very early time of infection, not many transcriptional differences were found. However, one gene was present as a large difference in both of the analyses: early growth response transcription factor 1 (*Egr1*), which encodes a transcription factor that mediates the immediate early response of cells to many stresses (Sukhatme, 1990). This gene is a likely candidate for upregulation by 1 h of infection, as *Egr1* is regulated at the transcriptional level, with message levels increasing within 30 min to 2 h after a stimulus (Yan et al., 2000). Interestingly, the gene for the chemokine CCL2 also was differentially expressed sufficiently to pass the criteria in the GeneSpring X analysis. It is a known transcriptional target of *Egr1* (e.g., Yan et al., 2000). These findings represent the earliest transcriptional effects associated with YopM and point to a possible direct effect of YopM in cells that may interact directly with *Y. pestis*.

**Table 6:** **Microarray results for 1-h infection, analyzed two ways^a^**.

**GenBank accession**	**Symbol**	**Name**	**Fold change, parent/Δ *yopM-1***
**GENESPRING X WITH LOWESS NORMALIZATION**			
NM_007913	*Egr1*	Early growth response protein 1	0.24
NM_008300	*Hspa4*	Heat shock protein 4; synonym, Apg-2	0.44
NM_008737	*Nrp*	Neuropilin-1	0.44
NM_011331	*Ccl12*	Cytokine (C-C motif) ligand 12	0.45
NM_011333	*Ccl2*	Cytokine (C-C motif) ligand 2	0.45
NM_010424	*Hfe*	Hemochromatosis	0.46
NM_008064	*Gaa*	Glucosidase, alpha, acid	0.50
NM_007457	*Ap1s1*	Adaptor protein complex AP-1, sigma 1	2.18
**BIOCONDUCTOR WITH R SOFTWARE**			
NM_173740	*Maoa*	Monoamine oxidase A	0.09
NM_133654	*Cd34*	CD34 antigen; sialomucin CD34	0.17
NM_007913	*Egr1*	Early growth response protein 1	0.17

a Data for ≥2-fold differences between responses to parent and ΔyopM-1 Y. pestis; current gene symbols and names obtained at MGI (Mouse Genome Informatics, http://www.informatics.jax.org/). The complete normalized log ratio data as analyzed by GeneSpring X are available at http://www.ncbi.nlm.nih.gov/geo/query/acc.cgi?acc=GSE41564

The transcriptional response by splenic CD11b^+^ cells infected for 18 h was remarkably restricted: only 176 of the 1561 gene targets on the chips showed changes in expression ≥2-fold compared to expression at 1 h p.i. 55 of these genes were in common between mice infected with the two *Y. pestis* strains, 97 were unique to mice infected with the YopM^+^ strain, and 24 were unique to mice infected with Δ*yopM-1 Y. pestis* (see full dataset at http://www.ncbi.nlm.nih.gov/geo/query/acc.cgi?acc=GSE41564). Table 7 shows those transcriptional responses that differed by ≥2-fold between mice infected with the two *Y. pestis* strains at 18 h. There was no agreement by the two data analyses, even for apparently strong differences (e.g., *Chi3l3*). Nonetheless, because the presence of YopM appeared to affect a few genes very strongly we repeated the 18-h infections and measured message abundance relative to the control gene *Ubc* for *Chi3l3* and *Cxcl2* and also the potentially relevant p47 Phox gene *Ncf1* in splenic CD11b^+^ cells by qRT-PCR. None differed by ≥2-fold for cells from mice infected with the two *Y. pestis* strains (data not shown). Taken together, these findings signify that there were no major transcriptional differences between CD11b^+^ cells from the two groups of mice at 18 h p.i.

**Table 7:** **Microarray results for 18-h infection, analyzed two ways^a^**.

**GenBank accession**	**Symbol**	**Name**	**Fold change, parent/Δ*yopM-1***
**GENESPRING X WITH LOWESS NORMALIZATION**			
NM_009892	*Chi3l3*	Chitinase 3-like 3	0.06
NM_009140	*Cxcl2*	Chemokine (C-X-C motif) ligand 2	0.17
XM_489530	*Igl-V1*	Immunoglobulin lambda variable 1	0.19
NM_010876	*Ncf1*	Neutrophil cytosol factor 1 (p47-phox)	0.25
NM_001033632	*Ifitm6*	Interferon-induced transmembrane protein 6	0.31
NM_009982	*Ctsc*	Cathepsin C	0.34
NM_008611	*Mmp8*	Matrix metallopeptidase-8	0.35
NM_010169	*F2r*	Coagulation factor II (thrombin) receptor	0.36
NM_029612	*Slamf9*	SLAM family member 9	0.38
NM_009735	*B2m*	Beta-2-microglobulin	0.39
NM_010583	*Itk*	Il2-inducible T cell kinase	0.43
NM_027592	*Taf9*	TAF9 RNA polymerase II, TATA box binding protein	0.43
NM_013563	*Il2rg*	Interleukin 2 receptor, gamma chain	0.43
NM_009684	*Apaf1*	Apoptotic protease activating factor 1	0.43
NM_009882	*Cebpz*	CCAAT/enhancer binding protein zeta	0.44
NM_009192	*Sla*	SRC-like-adapter (mSLAP)	0.44
NM_133948	*Psip1*	PC4 and SFRS1 interacting protein 1	0.44
NM_008394	*Irf9*	Interferon regulatory factor 9	0.44
NM_138952	*Ripk2*	Receptor (TNFRSF)-interacting serine/threonine-protein kinase 2	0.45
NM_016787	*Bnip2*	BCL2/adenovirus E1B interacting protein 2	0.45
NM_008390	*Irf1*	Interferon regulatory factor 1	0.46
NM_010480	*Hsp90aa1*	Heat shock protein 90, alpha (cytosolic) class A member 1	0.47
NM_010551	*Il16*	interleukin 16	0.47
NM_007452	*Prdx3*	Peroxiredoxin 3	0.47
NM_173740	*Maoa*	monoamine oxidase A	3.52
NM_007641	*Ms4a1*	Membrane-spanning 4-domains, subfamily A, member 1	3.14
NM_008339	*Cd79b*	CD79B antigen	2.42
**BIOCONDUCTOR WITH R SOFTWARE**			
NM_007440	*Alox12*	Arachidonate-12 lipoxigenase	0.16
NM_009715	*Atf2*	Activating transcription factor 2	0.15
NM_001163247	*Birc7*	Baculoviral IAP repeat-containing 7 (livin)	0.13
NM_009902	*Cldn3*	Claudin 3	0.14
NM_008326	*Irgm1*	Immunity-related GTPase family M member 1	0.24

a Data for ≥2-fold differences between responses to parent and ΔyopM-1 Y. pestis; current gene symbols and names obtained at MGI (Mouse Genome Informatics, http://www.informatics.jax.org/). The complete normalized log ratio data as analyzed by GeneSpring X are available at http://www.ncbi.nlm.nih.gov/geo/query/acc.cgi?acc=GSE41564

Table 8 shows that the microarray findings for *Egr1* and *Ccl2* at 1 h p.i. were reproduced in confirmation tests by qRT-PCR. The YopM-related effect on *Egr1* expression was a downregulation compared to the level in CD11b^+^ cells from non-infected mice. We also tested genes for three other proinflammatory chemokines/cytokines and found a small upregulation of *Il1β* in the absence of YopM. There were no significant differences in expression of these genes between CD11b^+^ cells from mice infected for 18 h with the two *Y. pestis* strains, consistent with the hypothesis that no major transcriptional changes related to YopM were occurring in this cell population at this time. However, these cells clearly were responding to the infection, as *Ccl2*, *Ccl3*, and *Il1β* were upregulated in expression compared to cells from non-infected mice.

**Table 8:** **Relative transcript levels^a^ for a set of inflammation-related genes in splenic CD11b^+^ cells from infected mice**.

	***Egr1***	***Tnf*α**	***Ccl2***	***Ccl3***	***IL1*β**
**Non-infected**	8.32 ± 2.11	1.07 ± 0.31	0.02 ± 0.01	0.73 ± 0.00	1.59 ± 0.03
**1-h INFECTION—CONFIRMATION TESTS^b^**					
Parent	1.38 ± 0.20	1.14 ± 0.70	0.12 ± 0.00	0.63 ± 0.19	1.33 ± 0.52
Δ*yopM-1*	9.40 ± 1.33^*^	1.70 ± 1.12	0.34 ± 0.05^***^	0.59 ± 0.06	2.88 ± 0.08^**^
Ratio P/M^c^	0.15		0.35		0.46
**18-h INFECTION^b^**					
Parent		2.20 ± 1.90	6.91 ± 1.58	13.02 ± 3.61	29.02 ± 16.23
Δ*yopM-1*		1.92 ± 2.51	3.88 ± 2.62	13.38 ± 8.37	7.24 ± 4.35

a *The data are the expression values normalized to that for Ubc calculated as 100(2^−(CPgene−CPUbc)^), where CP_Ubc_ is the geometric mean from all experiments. Each experiment was carried out at least twice, and the data are the averages ± SD*.

b *The confirmation tests for 1 h infections were done by qRT-PCR on the RNA that was used for microarray. The data for non-infected and 18-h infected splenic CD11b^+^ cells used RNA from independent sets of replicate experiments*.

c *Ratio P/M is the ratio of average relative gene expression for mice infected by parent Y. pestis to that for mice infected with the ΔyopM-1strain*.

* *Data for parent differed significantly from those for ΔyopM-1 at p < 0.05*.

** *Data for parent differed significantly from those for ΔyopM-1 at p < 0.005*.

*** *Data for parent differed significantly from those for ΔyopM-1 at p < 5 × 10^−6^*.

### Inflammatory gene expression using *in-vitro* infection models

In the interest of identifying a system to extend the studies to the biochemical level with a pure cell population, we tested whether BMMs infected *in-vitro* for 2 h would show YopM-related transcript differences at 6 h for the same genes examined in the experiments of Table 8. Table 9 shows that the BMMs responded to infection by upregulating expression of all five genes tested; but there were no YopM-related differences, even for *Ccl2*. *Egr1* in infected BMMs showed no difference from non-infected cells, as expected for this transiently expressed gene after 6 h infection.

**Table 9:** **Relative gene expression^a^ in cultured MΦs**.

**BMMs^b^**	***Egr1***	***Tnf*α**	***Ccl2***	***Ccl3***	***IL1*β**	**C*xcl1***
**Non-infected**	2.60 ± 0.06	2.71 ± 2.30	6.52 ± 2.56	36.0 ± 2.61	0.05 ± 0.01	0.08 ± 0.11
**2-h INFECTION FOLLOWED BY 4-h INCUBATION**						
Parent	2.83 ± 1.18	417 ± 82.6	665 ± 211	1730 ± 165	258 ± 34.7	9.04 ± 7.63
Δ*yopM-1*	2.52 ± 1.65	371 ± 36.4	674 ± 89.5	1580 ± 260	201 ± 26.4	9.11 ± 7.22
**BMMs^c^**						
**1-h INFECTION**						
Parent	2.23 ± 1.28					
Δ*yopM-1*	4.45 ± 4.37^*^					
**BMMs^d^**	***Egr1***	***Il6***	***Cxcl10***	***IL 10***		
**Non-infected**	5.9 ± 4.6	0.0 ± 0.0	19.2 ± 26.1	0.1 ± 0.0		
**1-h INFECTION**						
pYopM	194 ± 102	0.1 ± 0.0	66.5 ± 49.4	21.2 ± 18.2		
Vector	273 ± 140	0.0 ± 0.0	58.8 ± 40.8	15.2 ± 5.8		
**3-h INFECTION**						
pYopM	58.0 ± 25.6		535 ± 733	23.1 ± 12.0		
Vector	55.2 ± 15.0		486 ± 464	17.5 ± 3.3		
**6-h INFECTION**						
pYopM	22.6 ± 12.2	6.1 ± 11.3	150 ± 104	15.5 ± 12.0		
Vector	27.8 ± 10.9	3.7 ± 3.2	92.0 ± 32.1	14.6 ± 7.6		
**J774A.1 Cells^d^**	***Egr1***	***Il6***	***Cxcl10***	***Tnf*α**	***Ccl2***	***Ccl3***
**Non-infected**	0.40 ± 0.40	0.40 ± 0.83	15.3 ± 12.3	517 ± 247	772 ± 681	2183 ± 1643
**1-h INFECTION**						
pYopM	26.2 ± 32.6	4.8 ± 4.4		3400 ± 2080	75.4 ± 42.8	6560 ± 7900
Vector	7.2 ± 8.3^*^	3.9 ± 4.1		3810 ± 2480	67.9 ± 22.2	5560 ± 4650
**3-h INFECTION**						
pYopM	33.7 ± 18.7			5050 ± 2820		5900 ± 570
Vector	55.9 ± 19.0^*^			3750 ± 1720		5170 ± 549
**6-h INFECTION**						
pYopM		0.80 ± 1.1	1.9 ± 0.41			17,600 ± 23,700
Vector		0.20 ± 0.17	4.0 ± 1.8^*^			10,300 ± 11,500

a *The data are the expression values for the indicated genes normalized to that for Ubc calculated as 100(2^−(CPgene−CPUbc)^), where CP_Ubc_ is the geometric mean from all experiments. Each experiment was carried out at least twice, and the data are the averages ± SD*.

b *BMMs were infected with parent or ΔyopM-1 Y. pestis at an MOI of 10 in replicate experiments with at least duplicate infected cultures analyzed separately. In these experiments, the medium was replaced after 2 h of infection with medium containing 5% FBS + 50 μg mL^−1^ gentamicin and 40 μg mL^−1^ chloramphenicol to kill both extracellular and intracellular bacteria*.

c *BMMs were infected with parent or ΔyopM-1 Y. pestis at an MOI of 10 in replicate experiments with quadruplicate infected cultures analyzed separately. The cells were pretreated with, and infected in the presence of, cytochalasin D to prevent engulfment of the bacteria and maximize delivery of YopM*.

d *BMMs and J774A.1 cells were infected at an MOI of 30–40 with Y. pestis KIM8-3003.12 containing pYopM or the vector as indicated. The cells were pretreated with, and infected in the presence of, cytochalasin D to prevent engulfment of the bacteria and maximize delivery of YopM. Gene expression in BMMs was assayed in two to three experiments with two to four replicate cultures analyzed separately. For J774A.1 cells at 1 h, Egr1 and Ccl3 were measured in triplicate in seven experiments and Ccl3 was measured in six experiments; the data for 3 h came from three experiments. At 6 h, numbers of experiments were 5 (Ccl3), 3 (Il6), and 2 (Cxcl10). Genes in non-infected J77A.1 cells were measured in 8 (Egr1), 8 (Il6), 5 (Cxcl10), 7 (Tnfα), 6 (Ccl2), and 10 (Ccl3) experiments*.

* *Data for vector vs. pYopM infections differed significantly at *p* < 0.05*.

To test for a YopM-related difference in *Egr1* expression, BMMs were infected for 1 h (Table 9). In these experiments, the cultured macrophages were pretreated and infected in the presence of 2.5 μM cytochalasin D, which has been used previously in experiments to measure Yops delivery to J774A.1 cells (Cowan et al., 2005). This refinement in the protocol was made to prevent phagocytosis of some yersiniae and their loss from the population delivering YopM, since YopM is believed to be delivered primarily by extracellular bacteria (e.g., Skrzypek et al., 1998). These tests revealed a statistically significant 2-fold lower *Egr1* expression when YopM was present in the infecting *Y. pestis* and extended our transcriptional findings from infected mice to an *in-vitro* infection model.

In an attempt to elicit a stronger *in-vitro* transcriptional phenotype for YopM that could be pursued biochemically, we infected BMMs and also J774A.1 monocyte-like cells at a higher MOI (30–40) with *Y. pestis* KIM8-3003.12, which lacks all 6 effector Yops and also the surface protease Pla that can degrade Yops (Sodeinde et al., 1988). *yopM* was overexpressed *in trans* on pYopM, or the yersiniae contained only the empty vector. The bacteria were given a 1-h induction at 37°C to prime for rapid delivery of YopM upon cell contact while retaining most of the reactogenic lipid A form characteristic of growth at ambient temperature (Rebeil et al., 2004). This was to provide a proinflammatory stimulus against which the effect of YopM might be revealed, because YopM would normally be functioning in a proinflammatory environment *in-vivo*. In these experiments the use of cytochalasin D to prevent engulfment of the bacteria was crucial, because these yersiniae lacked the antiphagocytic Yops.

Table 9 shows the results. BMMs infected for 1 h showed strongly upregulated expression of *Egr1* and a trend toward lower expression when infected with the pYopM-containing *Y. pestis* KIM8-3003.12 compared to the vector-only control strain, but the effect was less than 2-fold and not statistically significant. As expected, *Egr1* expression had declined by 3 h and further by 6 h of infection. No YopM-related differences were found for the other genes tested.

In J774A.1 cells infected for 1 h with either *Y. pestis* strain, all genes tested except *Ccl2* were expressed at levels significantly greater than those in non-infected cells (highest P by *t*-test was <0.005) (Table 9). *Ccl2* expression was downregulated by infection with both YopM-expressing and control *Y. pestis* strains (*P* < 0.05). Interestingly, there were significant YopM-related differences in *Egr1* expression, with 3.6-fold greater expression when YopM was present at 1 h compared to cells infected with the vector-only strain (*P* < 0.05). This pattern had reversed by 3 h p.i. (*P* < 0.05), and expression was still strong, indicating that in these cells YopM can have both early and delayed effects on *Egr1*. No other genes tested showed YopM-related differences in expression at either 1 or 3 h. *Cxcl10*, which was assayed only after 6 h, was downregulated in expression by infection (*P* < 0.05 for both *Y. pestis* strains) but showed greater downregulation when YopM was present (*P* < 0.05).

## Discussion

In this study we sought to identify early effects of *Y. pestis* YopM *in-vivo* and characterize potential molecular pathways that YopM modulates. We used a high-dose infection model to influence as many of the direct target cells for *Y. pestis* as possible in their natural context while retaining the phenotype of the Δ*yopM* strain. The synchronous infection due to the intravenous route was then exploited in a series of molecular discovery assays. We chose 1 and 16 h p.i. for analysis as times when the two bacterial strains were present in the same numbers, but the Δ*yopM* strain had reached its peak and would not significantly increase thereafter due to the host response that normally is counteracted by YopM (Figure 1A). The spleens of the mice at 16 h p.i. had similar acute inflammatory foci at the periphery of many follicles. The numbers of live PMNs and inflammatory MOs were declining but still elevated, and cells obtained by immunomagnetic beads were viable.

### CD11b^+^ cells participate in the early host response to systemic plague

Although this model was not intended to replicate systemic plague that occurs following low-dose peripheral infection, it did provide the earliest available picture of host responses to systemic plague and important new findings. A recent study of the systemic phase of bubonic plague due to fully virulent *Y. pestis* CO92 showed high levels of the PMN maturation factor G-CSF, chemokines CXCL1 and CCL2, as well as cytokines IL-6 and Il-1α in plasma (Demeure et al., 2012). These responses occurred by day 2 after subcutaneous infection of rapid-responding plague-resistant SEG mice but were delayed in susceptible C57BL/6 mice. Systemic bacterial numbers were distinctively higher on day 2 in the SEG strain, and chemokine/cytokine response correlated with bacterial burden. In our study with a high dose given intravenously, we also obtained high levels of G-CSF, CXCL1, CCL2, and IL-6 in serum at 16 h p.i. in C57BL/6 mice (IL-1α was not tested) and this agreement indicated that findings from this model were not artifactual. Despite very high amounts of G-CSF in serum at 16 h p.i., GM-CSF, a maturation factor for MOs, DCs, and PMNs, was not detectable, and the MO maturation factor M-CSF was present only at low levels. The CSFs are produced by many cell types, including T cells, endothelial cells and stromal cells in addition to MΦs, and a circuit of amplification is hypothesized in which stimulated MΦs produce cytokines that in turn stimulate CSF production from surrounding cells (Hamilton, 2008). The basis of selective expression of G-CSF in infection is not fully understood, but it is part of the response to sepsis (e.g., Cebon et al., 1994) and is not unique to plague. A striking feature of our findings for sera at 16 h p.i. was the absence of the pro-inflammatory cytokine IL-1β, which also was not elevated in plasma or organs at any time in the study by Demeure et al. (2012). *Y. pestis* has been found to stimulate caspase-1 activation and IL-1β production in MΦs (e.g., Bergsbaken and Cookson, 2007; Lilo et al., 2008); however, this effect apparently was not dominant early in systemic plague.

We found a high level of the T helper 1 (Th1)-biasing cytokine IFNγ and very high levels of two chemokines that are involved in type 1 immune responses: CXCL9 and CXCL10 [not tested by Demeure et al. (2012)]. These observations could indicate that an early response to high numbers of *Y. pestis* includes mobilization of a Th1-biased host response in addition to innate defenses, consistent with the absence of elevated levels of the Th2 cytokines IL-4 and IL-10 in sera. However, these chemokines increasingly are being recognized as part of the early response to acute microbial infections. Similarly to defensins, they can serve direct anti-microbial roles against Gram-negative pathogens (Cole et al., 2001), they are expressed early and promote bacterial clearance of *Klebsiella pneumoniae* in experimental pneumonia (Zeng et al., 2005), and they can promote survival against sepsis by stimulating enhanced PMN recruitment and phagocytic activity (Kelly-Scumpia et al., 2010). They may have protective potential against systemic plague.

The chemokines/cytokines in sera represent the pooled output of leukocytes and tissue cells in both liver and spleen, and there were no large differences between sera of mice infected with YopM^+^ compared to Δ*yopM-1 Y. pestis*. To focus on the direct effects of YopM on cells to which *Y. pestis* delivers Yops, we measured chemokines/cytokines produced by a subpopulation of splenic leukocytes. *Y. pestis* KIM5 was previously found to deliver a tagged YopM selectively to CD11b^+^ CD11c^−^ MΦs, CD11c^+^ CD11b^−^ DCs, and Gr1^+^ cells (PMNs, MOs, and iDCs), but not to CD4^+^ or CD8^+^ T cells or CD19^+^ B cells in spleens of C57BL/6 mice (Marketon et al., 2005). We previously showed that Ly6G^+^ PMNs are not required for the pathogenic effect of YopM in spleens of C57Bl/6 mice infected with parent *Y. pestis* KIM5 compared to the Δ*yopM-1* strain (Ye et al., 2011). Accordingly, we removed B, T, and dead cells along with Ly6G^+^ PMNs from the splenic homogenates prepared from mice infected for a short time with YopM^+^ or Δ*yopM-1Y. pestis*, and recovered CD11b^+^ cells for analysis. Surprisingly, a third of the resulting cells, even from non-infected mice, had the appearance of immature PMNs. These cells were released by the Miltenyi dissociator protocol used in these experiments: fewer such cells were present in the populations that we obtained for studies of gene expression with what may be a gentler homogenization method. Nonetheless, they were present in all of the mice and could have participated in cytokine production. At 16 h p.i. the CD11b^+^ cell population likely did contribute to the high levels of CXCL9, CXCL10, and IFNγ that were present in mice infected with either YopM^+^ or Δ*yopM-1 Y. pestis*. Further, the gene for CXCL10 was strongly induced after 6 h p.i. in total splenic leukocytes from SCID mice infected with a high but non-lethal dose of *Y. pestis* lacking effector Yops or expressing only YopM (R. L. Chelvarajan and S. C. Straley, unpublished data). These findings provided additional support to the idea that mice with systemic plague mobilize a Th1-biased innate response early in infection. The data also indicated that CD11b^+^ cells contribute to this response; but they did not point to a major role for YopM in early chemokine/cytokine production.

### The early transcriptional response by CD11b^+^ cells in systemic plague

Our microarray experiments tested whether the presence of YopM affects expression of more than 1500 genes early in CD11b^+^ cells from infected spleens. In these tests MOs/MΦs constituted more than two thirds of the CD11b^+^ population that we obtained. Overall, the transcriptomic response was limited in scope, reflecting the remarkably stealthy character of even high numbers of *Y. pestis*. This phenomenon was noted in the microarray study of gene expression in lymph nodes of rats with bubonic plague (Comer et al., 2010). We found no genes with YopM-associated significant changes in expression at 18 h p.i. There was, however, a YopM-associated downregulation of the gene for *Egr1*, at 1 h p.i. This is the earliest time examined so far for *Y. pestis* infection *in-vivo* and was possible because of the synchronous infection used in this work. *Egr1* is expressed in response to a wide range of cellular stresses, including bacterial and parasitic infection (De Grado et al., 2001; Wiley et al., 2011), and its product contributes to the regulation of hundreds of genes. It was identified as a member of a “core response module” in macrophages consisting of 38 genes that all show differential responses to stimuli and that tend to be targeted by pathogens (McDermott et al., 2011). Proinflammatory genes that are upregulated by the binding of *Egr1* to their promoters encode TNFα, tissue factor, ICAM-1, CCL2, IL-8, and IL-6 (Guha et al., 2001; Shi et al., 2002; Hoffmann et al., 2008). Accordingly, we included *Tnf*α, *Ccl2*, and *Il6* among the genes evaluated with *in-vitro* infection models.

### YopM-associated gene expression in BMMs and J774A.1 cells

We wanted to determine if altered gene expression due to the presence of YopM could be detected in an *in-vitro* infection model with a single host cell type. Macrophages were chosen because of their predominance in the populations studied by microarray and because of the predicted central role of *Egr1* in their responses. Recent studies have found that YopM can spontaneously penetrate cells and cause ca. 2-fold downregulation of *Tnf*α expression in HL60-derived macrophages by 6 h following exposure to a high concentration (25 μg ml^−1^) of pure protein (Rüter et al., 2010). YopM also could significantly counteract the stimulatory effect of LPS on mRNA levels for *Tnf*α and several other cytokine genes in these cells (assayed after 16 h; Rüter et al., 2010). Therefore, we revisited macrophage cell-culture infection models to determine if delivery of YopM by infection was downregulatory on gene expression. Despite using multicopy expression of *yopM*, a high MOI, inhibition of phagocytosis to retain strong T3SS delivery by extracellular bacteria, and the absence of other Yops that would compete for the T3SS channel or downregulate NF-κB and MAP kinase signaling, we found only small downregulatory effects of YopM on *Egr1* expression in BMMs. However, J774A.1 cells did show significant YopM-related early transcriptional responses. BMMs have a mature macrophage phenotype, whereas J774A.1 cells are intermediate MO-MΦ-like cells (Chamberlain et al., 2009), so it was reasonable to test them as a model for cells from an early inflammatory focus. These cells showed a biphasic response by *Egr1* to the presence of YopM, being initially upregulated compared to cells infected without YopM delivery and then comparatively downregulated at 3 h p.i. This is intriguing and raises the possibility that YopM has distinct effects depending on its state of trafficking in the cell, as little YopM is observed in the nucleus by 1 h p.i., but a significant amount can be there by 3 h (Skrzypek et al., 1998). We found no significant YopM-related differences in *Tnf*α message levels at 3 h p.i., perhaps because of the opposing effects on *Egr1*. It is meaningful to test for *Tnf*α message this early in infection, because it can be induced within an hour in response to stimuli (Espel et al., 1996; Raabe et al., 1998), and a significant downregulating effect was observed at 3 h by Rüter et al. (2010) in HL60-derived macrophages treated with pure YopM. The other tested gene that was differentially affected by the presence of YopM was *Cxcl10* at 6 h p.i. This gene is not known to be directly regulated by *Egr1*; however, a potential effect of YopM on *Cxcl10* expression could be real and worthy of further investigation, because CXCL10 levels in serum were slightly lower at 16 h p.i. in mice infected by the YopM^+^ strain. Our findings from J774A.1 cells are consistent with an effect of YopM at the level of transcription early in infection.

### Integration of these findings with the growing picture of YopM function at the biochemical level

*Egr1* transcription is activated in response to MAPK pathways that lead to activation of the transcription factor Elk-1, such as the Ras-Raf-MEK-ERK1/2 pathway (Guha et al., 2001). For example, in response to LPS, ERK1/2-activated Elk-1, along with serum response factor (SRF), bind and activate the *Egr1* promoter in THP-1 human monocytic leukemia cells (Guha et al., 2001). The nuclear-localized transcription factor SRF is a substrate of RSK1, and phosphorylation of SRF by activated RSK1 increases the affinity of SRF for serum response elements in promoters (Romeo et al., 2012) such as that for *Egr1*. YopM's interaction with RSK1 might downregulate *Egr1* indirectly by forming large complexes with RSK1 in the cytosol, thereby sequestering this kinase there, and effectively preventing its access to the nucleus and to SRF. Decreased levels of *Egr1* could result in reduced transcription of *Egr1* target genes such as *Tnf*α, as seen by Rüter et al. (2010). RSK1 is also involved in regulation of IFNγ-dependent translation (Kroczynska et al., 2011); however, such a role was not evident at 16 h of infection, when we characterized chemokines/cytokines present in serum and produced by CD11b^+^ cells. We believe that our findings link YopM's binding of RSK1 to indirect early effects on transcription.

However, binding to RSK1 does not account for all of YopM's pathogenic effect, because a YopM that was unable to bind RSK1 still endowed *Y. pseudotuberculosis* with more pathogenic potential than that of a strain without YopM (McCoy et al., 2010). PRK2 binds at a more internal site on YopM than does RSK1 and could be involved (McPhee et al., 2010). Further, YopM might interact with numerous cellular components during its trafficking, with potential pathogenic consequences (Skrzypek et al., 1998); and YopM does go to the nucleus and likely does function there.

### The larger picture of YopM function

YopM is a major virulence factor in systemic plague due to Δ*pgm Y. pestis* in C57BL/6 mice; yet its phenotype *in-vivo* is not manifested until between days 1 and 2 after intravenous infection. Even in the present study with a dose of *Y. pestis* that was more than a 1000-fold higher than used previously, there was a delay in appearance of the growth-related phenotype. These observations suggest that YopM's target may be a component of the host response that requires time to develop, and that until this response occurs, YopM does not have a major role at the level of transcription. YopM's potential early effects such as subtle downregulation of *Tnf*α in resident cells are not likely to be YopM's major function, due to redundancy to other Yops that downregulate NF-κ B and MAP kinase pathways as well as the activity of the *Y. pestis* anti-inflammatory modified lipid A.

The difficulty in establishing *in-vitro* models for dissecting YopM's major effects could in part arise from failure to replicate the inflammatory character of the environment and/or cells in which YopM functions. Perhaps that is why we obtained a greater YopM-related response in immortalized monocyte-like cells rather than BMMs, and why Rüter et al. (2010), by using LPS stimulation of immortalized HL60-derived MΦs and waiting 16 h, found downregulatory effects on cytokine genes as diverse as *TNF*α, *IL18, IL12p35*, and *Il15*. The transcriptional effects seen by Rüter et al. (2010) were relatively small, of the order of 2-fold or less. However, other stimulations of MO/MΦ-lineage cells might create target cells in which YopM shows a strong effect.

Infection of mice with YopM-producing *Y. pestis* caused a global effect in spleen that could protect the Δ*yopM-1* strain *in trans* by preventing the stable accumulation of iDCs recruited through CCR2 (Ye et al., 2011). There was an initial influx of monocytes during the first 24 h in spleens of mice infected with YopM^+^ *Y. pestis*, but by day 2 p.i. these cells as well as NK cells were disappearing (Kerschen et al., 2004; Ye et al., 2011). This means that at days 2 and 4 p.i. when RNA levels were measured previously (Kerschen et al., 2004), the spleens infected with the two *Y. pestis* strains contained qualitatively different cell populations, and whether YopM caused the differences in transcription is not known. In contrast, in liver there was no YopM-associated recruitment effect, the YopM^+^ strain did not protect *in trans*, and PMN function was implicated as being targeted directly or indirectly by YopM (Ye et al., 2011). Nonetheless, the YopM-associated growth difference between parent and Δ*yopM-1* strains occurs simultaneously in liver and in spleen. We hypothesize that YopM's molecular targets are the same in the two organs even though there are organ-specific cellular responses. YopM may have its primary role on activated cells or may drive a local MDSC response (Haverkamp, 2011), and transcriptional effects in these cells may be downregulated directly or indirectly.

### Conflict of interest statement

The authors declare that the research was conducted in the absence of any commercial or financial relationships that could be construed as a potential conflict of interest.
